# Children’s externalizing and internalizing symptoms and their involvement in decision-making

**DOI:** 10.1192/j.eurpsy.2022.1809

**Published:** 2022-09-01

**Authors:** B. Németh, M. Miklósi

**Affiliations:** 1 Eotvos Lorand University, Doctoral School Of Psychology, Budapest, Hungary; 2 Hintalovon Child Rights Foundation, Na, Budapest, Hungary; 3 Eötvös Loránd University, Department Of Developmental And Clinical Child Psychology, Budapest, Hungary; 4 Heim Pál Children’s Hospital, Mental Health Centre, Budapest, Hungary

**Keywords:** decision-making, externalizing and internalizing symptoms, child and youth, participation

## Abstract

**Introduction:**

The involvement of children in decision making processes was shown to have beneficial effects on their cognitive, emotional, and social development. However, no research focused on its association with child’s psychopathology.

**Objectives:**

Our research aimed to explore the relationships between children’s externalizing and internalizing symptoms and their involvement in decision making in a dimensional approach.

**Methods:**

A community sample of 318 parents (64.2% mothers, mean age: 39.48 years SD=5.82) filled out an online questionnaire including the Decision-Making Involvement Scale (DMIS) assessing the parent’s and child’s behaviour in decision-making processes and the Strength and Difficulties Questionnaire (SDQ). Linear regression analyses were conducted for exploring multivariate associations of DMIS Parent and Child subscales with child’s psychopathology and prosocial behaviour, controlling for child’s gender and age.

**Results:**

With SDQ Internalizing problems subscale as dependent, linear regression analysis did not result in a significant model. In a significant model explaining 21.2% of the variance of the dependent variable, SDQ Externalizing problems score were significantly related to child’s age and gender, and to both Child and Parent subscales of the DMIS. When choosing SDQ Prosocial behaviour subscale as dependent, child’s gender and DMIS Child subscale were significantly associated to the dependent variables, accounting for 12.2% of the variance.

**Conclusions:**

Our results suggest that children’s involvement in decisions may be related to less externalizing symptoms and higher levels of prosocial skills. However, longitudinal research is needed to uncover the direction of the relationship and underlying mechanisms.

**Disclosure:**

No significant relationships.

